# Luminescent Iridium(III) Complexes Supported by N-Heterocyclic Carbene-based C^C^C-Pincer Ligands and Aromatic Diimines

**DOI:** 10.1038/srep15394

**Published:** 2015-10-21

**Authors:** Lai-Hon Chung, Hoi-Shing Lo, Sze-Wing Ng, Dik-Lung Ma, Chung-Hang Leung, Chun-Yuen Wong

**Affiliations:** 1 Department of Biology and Chemistry, City University of Hong Kong, Tat Chee Avenue, Kowloon, Hong Kong SAR; 2 State Key Laboratory of Millimeter Waves, City University of Hong Kong, Tat Chee Avenue, Kowloon, Hong Kong SAR; 3 Department of Chemistry, Hong Kong Baptist University, Kowloon Tong, Hong Kong SAR; 4 State Key Laboratory of Quality Research in Chinese Medicine, Institute of Chinese Medical Sciences, University of Macau, Macao, China

## Abstract

Iridium(III) hydrido complexes containing N-heterocyclic carbene (NHC)-based pincer ligand 1,3-bis(1-butylimidazolin-2-ylidene)phenyl anion (C^1^^C^C^1^) or 1,3-bis(3-butylbenzimidazolin-2-ylidene)phenyl anion (C^2^^C^C^2^) and aromatic diimine (2,2′-bipyridine (bpy), 1,10-phenanthroline (phen), 4,4′-dimethyl-2,2′-bipyridine (Me_2_bpy), or dipyrido-[3,2-f:2′,3′-h]-quinoxaline (dpq)) in the form of [Ir(C^C^C)(N^N)(H)]^+^ have been prepared. Crystal structures for these complexes show that the Ir–C_NHC_ distances are 2.043(5)–2.056(5) Å. The hydride chemical shifts for complexes bearing C^1^^C^C^1^ (−20.6 to −20.3 ppm) are more upfield than those with C^2^^C^C^2^ (−19.5 and −19.2 ppm), revealing that C^1^^C^C^1^ is a better electron donor than C^2^^C^C^2^. Spectroscopic comparisons and time-dependent density functional theory (TD-DFT) calculations suggest that the lowest-energy electronic transition associated with these complexes (λ = 340–530 nm (ε ≤ 10^3^ dm^3^ mol^−1^ cm^−1^)) originate from a d_π_(Ir^III^) → π*(N^N) metal-to-ligand charge transfer transition, where the d_π_(Ir^III^) level contain significant contribution from the C^C^C ligands. All these complexes are emissive in the yellow-spectral region (553–604 nm in CH_3_CN and CH_2_Cl_2_) upon photo-excitation with quantum yields of 10^−3^–10^−1^.

Polypyridyl ruthenium(II) or other d^6^-transition metal complexes represent an important class of emissive molecular material^1^,^2^,^3^,^4^,^5^,^6^,^7^,^8^,^9^,^10^,^11^,^12^,^13^,^14^. Their triplet [d_π_(M) → π*(polypyridyl)] metal-to-ligand charge transfer (^3^MLCT) excited-states are known to derive rich photophysical and photochemical properties, and their applications in solar energy conversion^11^,^15^, organic light emitting devices (OLEDs)^16^, photochemistry^11^,^12^, and bio-labelling reagents^17^ have received considerable interest. Regarding the design of transition metal-containing luminophores, iridium(III) center has received great attention on the basis that it is a heavier analogue of ruthenium(II) center^18^,^19^.

After the isolation of stable NHCs by Arduengo and co-workers in 1991^20^, intensive investigations on NHCs and the derived metal complexes have been centralized on the development of catalytic reagents for organic transformations^21^,^22^,^23^,^24^,^25^. On the other hand, employment of N-heterocyclic carbenes (NHCs)-derived ligands as an alternative of polypyridines in the design of transition metal-based luminophores is growing to be an important research topic recently. For example, emissive Ru(II)^26^, Ir(III)^18^,^19^, and Pt(II)^27^ complexes supported by NHCs have been reported. Several emissive NHC-containing multinuclear Cu, Ag, and Au complexes have also been prepared, in which the NHCs facilitate the metal-metal interaction-induced emissions^28^,^29^.

We have initiated a program to develop organometallic Ru(II)/Os(II)–diimine and related luminophores^30^,^31^,^32^,^33^, and very recently we have reported emissive osmium(II) carbonyl complexes bearing 1,3-bis(1-methylimidazolin-2-ylidene)phenyl anion (^Me^C^1^^C^C^1Me^) or 1,3-bis(3-methylbenzimidazolin-2-ylidene)phenyl anion (^Me^C^2^^C^C^2Me^) and aromatic diimine in the form of [Os(C^C^C)(N^N)(CO)]^+^^30^. Spectroscopic and theoretical investigations on [Os(C^C^C)(N^N)(CO)]^+^ have revealed that the emissive state for [Os(C^C^C)(N^N)(CO)]^+^ originates from a d_π_(Os^II^) → π*(N^N) MLCT transition, where the C^C^C ligands contribute significantly to both the d_π_(Os^II^) and π*(N^N) levels. This suggests that the NHC-derived ligands would not only act as point charge/spectator ligands, but can also be involved in the emissive excited-state to modify the photophysical properties of a metal–diimine luminophore. As an extension to scrutinize the effect of C^C^C pincer ligands on the photophysical properties of a [M(N^N)] moiety, we now present the preparation, spectroelectrochemical, photophysical, and theoretical investigations of a class of emissive hydrido iridium(III) complexes bearing C^C^C pincer ligands and aromatic diimines, [Ir(C^C^C)(N^N)(H)]^+^.

## Results

### Synthesis

**Figure 1:**
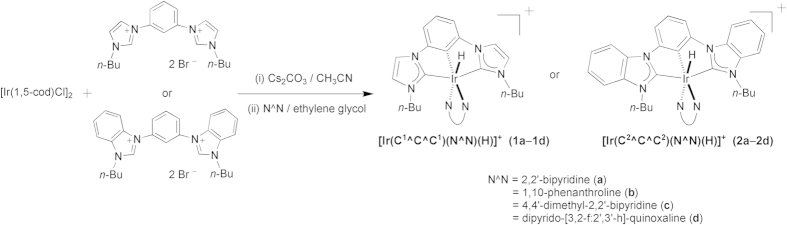
Synthetic route of 1–2

Emissive Ir(III) complexes [Ir(C^C^C)(N^N)(H)]^+^ (**1**–**2**) were prepared by refluxing [Ir(C^C^C)(CH_3_CN)(Br)(H)] with N^N in ethylene glycol (Figure 1). [Ir(C^C^C)(CH_3_CN)(Br)(H)] were synthesized analogously to the corresponding known complexes [Ir(^Ar^C^C^C^Ar^)(CH_3_CN)(Cl)(H)] and [Ir(^Me^C^1^^C^C^1Me^)(CH_3_CN)(I)(H)] (^Ar^C^C^C^Ar^ = 1,3-bis(1-arylimidazolin-2-ylidene)phenyl anion)^23^,^24^. The presence of the hydride ligands in **1**–**2** was confirmed by the ^1^H NMR signals at -20.6 to -19.2 ppm and ν_Ir–H_ at 2126 to 2189 cm^−1^. Both the ^1^H and ^13^C NMR spectra signify that **1**–**2** possess a pseudo-plane of symmetry in solution on the NMR time scale at room temperature. For instances, there are 17 and 19 sets of aromatic ^13^C signal for **1a** and **1b**, respectively. The ^13^C NMR signals at 167.8–180.6 ppm for **1–2** are typical for metalated NHC. It is noted that the hydride chemical shifts for **1a**–**1d** (-20.55 to -20.27 ppm) are nearly 1 ppm more upfield than those for **2a**–**2d** (-19.50 to -19.21 ppm). Since the hydride chemical shifts indicate that the electronic shielding effect of the hydrido group results from the metal core’s electron cloud, they can be used as probes for the donating ability of the C^C^C ligands^34^. Therefore, the more upfield hydride chemical shifts for **1a**–**1d** when compared with **2a**–**2d** reveals that C^1^^C^C^1^ is a stronger electron donor than C^2^^C^C^2^. The same conclusion has recently been made in the comparison of the ν_CO_ between [Os(^Me^C^1^^C^C^1Me^)(N^N)(CO)]^+^ and [Os(^Me^C^2^^C^C^2Me^)(N^N)(CO)]^+^ 31. These findings are also consistent with the NHC donor strengths determined by Huynh *et al*., where benzimidazolin-2-ylidene is suggested to have a weaker donor strength compared with imidazolin-2-ylidene^35^. Complex [Ir(C^1^^C^C^1^)_2_]^+^ has also been synthesized according to the method reported in literature^23^ for spectroscopic comparisons.

**Figure 2:**
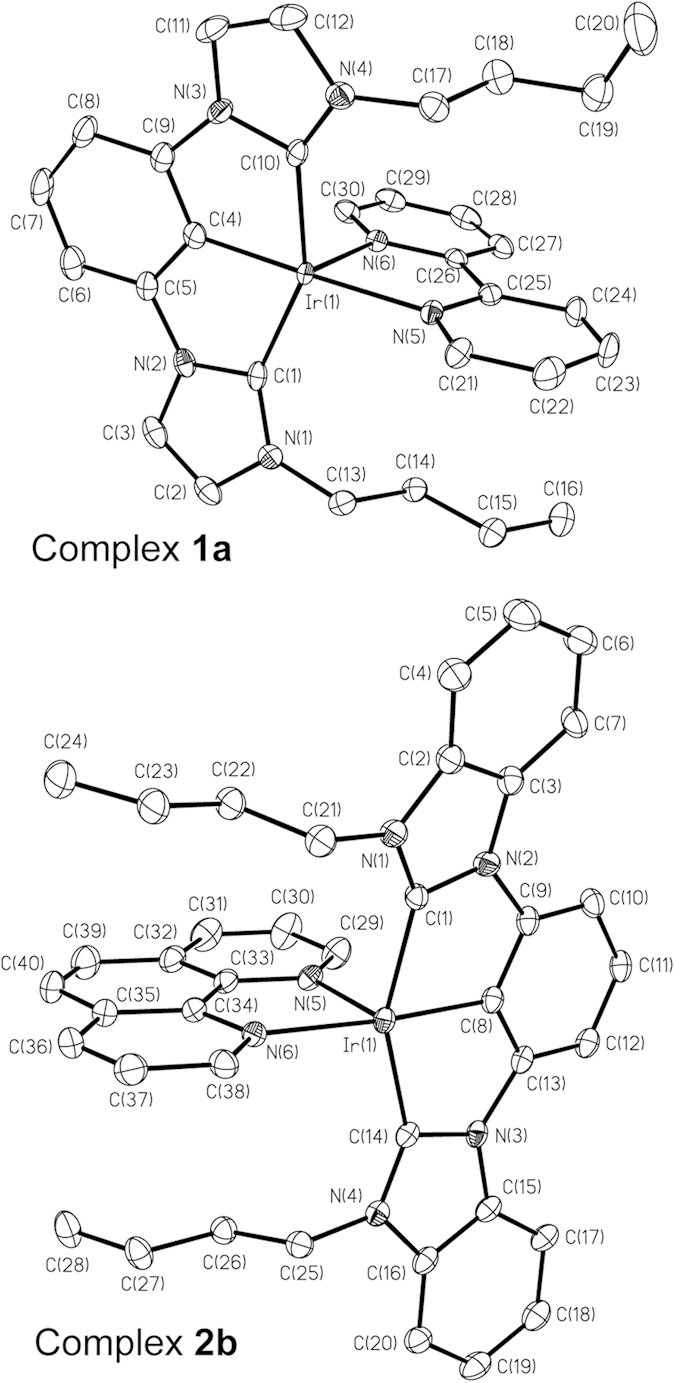
Perspective views of **1a** (top) and **2b** (bottom); thermal ellipsoids are at the 50% and 30% probability level, respectively. Hydrogen atoms are omitted for clarity.

The molecular structures of **1a**(ClO_4_), **2a**(ClO_4_), and [**2b**(ClO_4_)]_3_·CH_3_CN have been determined by X-ray crystallography. Perspective views of the cations **1a** and **2b** are depicted in Figure 2; selected bond distances and angles are summarized in Table 1. In each case, the Ir atom adopts a distorted octahedral geometry, with the C^C^C-pincer coordinating in a meridional mode. The ring systems on C^C^C are not perfectly co-planar: the NHC moieties (i.e. imidazolin-2-ylidene or benzimidazolin-2-ylidene units) are tilted towards the hydride ligands, and the angles between the NHC planes are 12.36–22.54°. These angles are larger than those found in [Ir(^Me^C^1^^C^C^1Me^)(CH_3_CN)(I)(H)], [Ir(C^^Me^C^Me^^C)(CH_3_CN)(I)_2_] (C^^Me^C^Me^^C = 1,3-bis(1-butylimidazolium)-4,6-dimethylbenzene) and [Ir(^Ar^C^C^C^Ar^)(CH_3_CN)(Cl)(H)] in which the angles between the NHC planes are 2.73°, 3.63° and 5.60–15.58° respectively^23^,^24^. As a comparison, the ring systems on C^C^C for [Os(^Me^C^1^^C^C^1Me^)(N^N)(CO)]^+^ and [Os(^Me^C^2^^C^C^2Me^)(N^N)(CO)]^+^ (N^N = bpy or phen) are more close to a co-planar configuration (angles between the NHC planes are 2.30–13.00°)^31^. The C_NHC_–Ir–C_Ph_ angles for these complexes are 77.22(11)–78.74(15)°, which are only slightly larger than the C_NHC_–Os–C_Ph_ angles in [Os(^Me^C^1^^C^C^1Me^)(N^N)(CO)]^+^ and [Os(^Me^C^2^^C^C^2Me^)(N^N)(CO)]^+^ (75.6(3)–76.8(3)°)^31^. The Ir–C_NHC_ distances (2.043(5)–2.056(5) Å) are notably longer than the Ir–C_Ph_ distances (1.959(4)–1.986(5) Å). Similar findings have been observed in [Ir(^Me^C^1^^C^C^1Me^)(CH_3_CN)(I)_2_]^23^, [Ir(^Me^C^1^^C^C^1Me^)(CH_3_CN)(I)(H)]^24^, [Ir(C^^Me^C^Me^^C)(CH_3_CN)(I)_2_] (C^^Me^C^Me^^C = 1,3-bis(1-butylimidazolium)-4,6-dimethylbenzene)^23^, [Ir(^Ar^C^C^C^Ar^)(CH_3_CN)(Cl)(H)]^24^, and Zr, Rh, and Os complexes bearing similar C^C^C-pincer ligands^31^,^36^. Since the Ir–C_Ph_ distances in *fac*-[Ir(C^C)_3_] and *mer*-[Ir(C^C)_3_] (C^C = 1-phenyl-3-methylbenzimidazolin-2-ylidene-*C*,*C*^2′^) are in the range of 2.071(7)−2.099(4) Å^18^, the significantly shorter Ir–C_Ph_ distances in this work most likely arise from the strain intrinsic to the metal–C^C^C moieties.

### Electrochemistry

Cyclic voltammetry has been used to examine the electrochemistry of the complexes (Table 2; all values vs Cp_2_Fe^+/0^). **1**–**2** show irreversible first oxidation waves at *E*_pa_ = 0.69 to 0.77 V (scan rate = 100 mV s^−1^), and reversible first reduction couples at *E*_1/2_ = -2.00 to -1.73 V. It is noted that both the first oxidation waves and the first reduction couples are sensitive to the change of C^C^C and N^N. For example, the first reduction potentials for **1a**–**1d** (-2.00 to -1.83 V) are slightly more negative than those for **2a**–**2d** (-1.95 to -1.73 V), and the ease of reduction follows the order: **d **> **b** ≈ **a **> **c**. These findings suggest that both the highest occupied molecular orbitals (HOMOs) and the lowest unoccupied molecule orbitals (LUMOs) for **1**–**2** contain contributions from the C^C^C and N^N, in agreement with our DFT calculations (see discussion below). Moreover, the contribution of N^N to the LUMOs of **1**–**2** is apparent as [Ir(C^1^^C^C^1^)_2_]^+^ does not feature any reduction wave within the solvent window.

### UV–Visible Absorption and Spectroelectrochemistry

**Figure 3:**
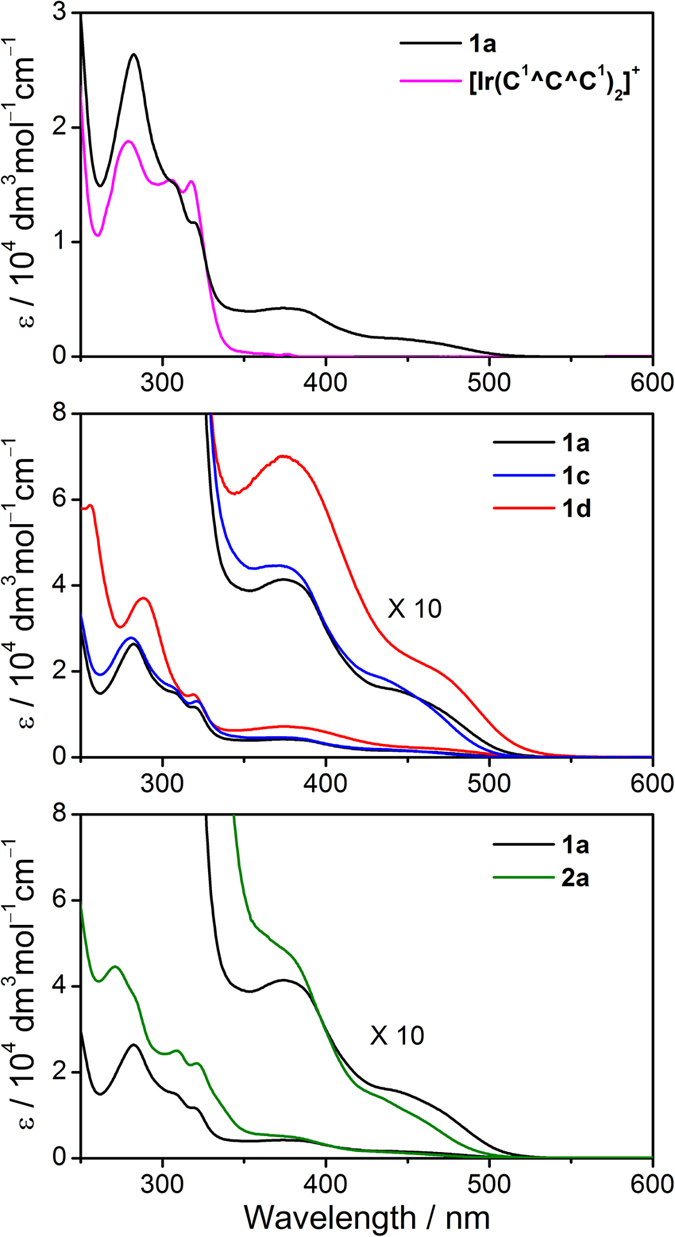
UV–visible absorption spectra of selected complexes in CH_3_CN at 298 K.

The UV–visible spectral data for **1**, **2**, and [Ir(C^1^^C^C^1^)_2_]^+^ are summarized in Table 3, and their absorption spectra are depicted in Figure 3. **1–2** exhibit intense, high-energy absorptions at λ ≤ 340 nm (ε ≥ 10^4^ dm^3^ mol^−1^ cm^−1^), and moderately intense bands at λ > 340 nm (ε ≈ 10^3^ dm^3^ mol^−1^ cm^−1^) with tailing up to 530 nm. In the literature, Ir(III) complexes bearing aromatic diimine ligands such as [Ir(bpy)_3_]^3+^ and [Ir(phen)_3_]^3+^ feature highly intense absorptions at λ ≤ 320 nm (ε ≥ 10^4^ dm^3^ mol^−1^ cm^−1^), and these are ascribed to π → π*(N^N) intraligand (IL) transitions^37^,^38^,^39^. In addition, [Ir(C^1^^C^C^1^)_2_]^+^ exhibits intense absorptions at λ ≤ 330 nm (ε ≥ 10^4^ dm^3^ mol^−1^ cm^−1^), which are expected to be a mixture of d_π_(Ir^III^) → π*(C^C^C) metal-to-ligand charge transfer (MLCT) and π → π*(C^C^C) IL transition. With the origin of absorptions for [Ir(N^N)_3_]^3+^ and [Ir(C^1^^C^C^1^)_2_]^+^ as references, the high-energy absorptions at λ ≤ 340 nm for complexes **1**–**2** are assigned to be a mixing of π → π*(C^C^C) IL, π → π*(N^N) IL, and d_π_(Ir^III^) → π*(C^C^C) MLCT transitions.

**Figure 4:**
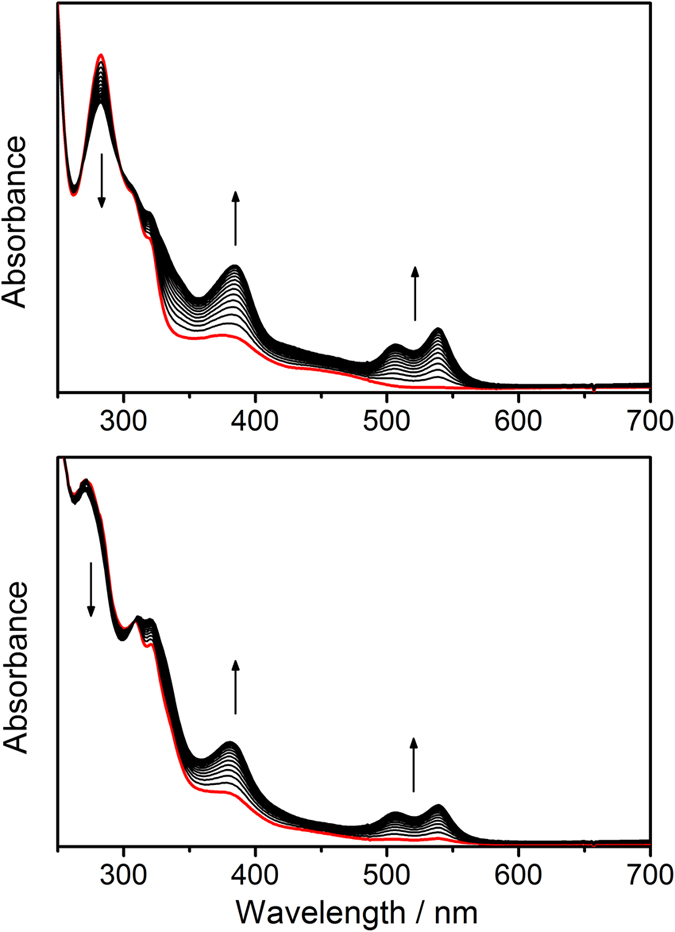
UV–visible absorption spectra for 1a (top) and 2a (bottom) in CH_3_CN at 298 K during electrochemical reduction at -1.95 V vs Cp_2_Fe^+/0^ (10 s traces; initial trace is shown in red).

On the other hand, the electronic transitions at λ = 340–530 nm (ε ≤ 10^3^ dm^3^ mol^−1^ cm^−1^) for **1**–**2** should contain some d_π_(Ir^III^) → π*(N^N) MLCT character, reasons are as follows: (1) [Ir(bpy)_3_]^3+^, [Ir(phen)_3_]^3+^, and [Ir(ppy)_2_(bpy)]^+^ (ppy = 2-phenylpyridine) feature d_π_(Ir^III^) → π*(N^N) MLCT transitions in similar energy region (λ_max_ = 370–520 nm, ε ≤ 10^3^ dm^3^ mol^−1^ cm^−1^);^38^,^39^,^40^,^41^,^42^,^43^ (2) a red-shift in absorption energy is observed when N^N is changed from Me_2_bpy to bpy, and from phen to dpq; (3) **1**–**2** display solvatochromic effect in the spectral region concerned. For example, the λ_max_ for **1a** within this spectral region is 374 nm in CH_3_CN, and is 384 nm in CH_2_Cl_2_; (4) there are no corresponding absorption bands for [Ir(C^1^^C^C^1^)_2_]^+^. This assignment is consistent with the TD-DFT calculations on complexes **1a** and **2a**, which suggest that the nature of electronic transitions in the spectral region concerned to be mainly attributed to the HOMO–1 → LUMO and HOMO–2 → LUMO transitions, where the HOMO–1 and HOMO–2 have higher Ir contribution (27–59%) than that in LUMO (3–4%), and LUMO has higher N^N contribution (93%) than those in HOMO–1 and HOMO–2 (3–15%) (see discussion below). The contribution of N^N to the LUMOs for **1** and **2** is further confirmed by spectroelectrochemistry. Thin-layer UV–visible spectroelectrochemistry has been employed to acquire the absorption spectra for **1a**^−^ and **2a**^−^, the reduced forms of **1a** and **2a** respectively (Figure 4). The isosbestic spectral changes suggest that the electrochemical reductions of **1a** and **2a** are clean conversions. Notably, reductions of **1a** and **2a** result in enhancement of absorption at ~380 nm and new absorption doublet band near 500 nm. These absorption features were observed in the reduction of [Ir(bpy)_3_]^3+^ and are characteristic absorptions for anionic bpy radical (bpy^•−^)^44^.

### Emission Spectroscopy

**Figure 5:**
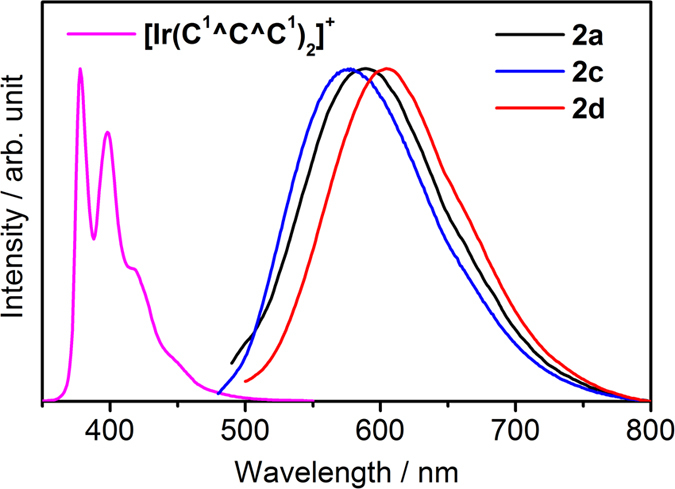
Emission spectra for 2a, 2c, 2d, and [Ir(C^1^^C^C^1^)_2_]^+^ (λ_ex_ = 420 nm for 1–2, 340 nm for [Ir(C^1^^C^C^1^)_2_]^+^).

The emission properties of the complexes in fluid solution (CH_3_CN and CH_2_Cl_2_) at 298 K have been investigated (Table 4). Figure 5 depicts the emission spectra for **2a**, **2c**, **2d**, and [Ir(C^1^^C^C^1^)_2_]^+^ in CH_3_CN at 298 K. Emission maxima of **1**–**2** range from 553 to 604 nm in CH_3_CN and CH_2_Cl_2_ which are significantly blue shifted when compared with [Os(C^C^C)(N^N)(CO)]^+^ (λ_em_ = 676–731 nm, solvents = CH_3_CN and CH_2_Cl_2_)^30^. Quantum yields (Φ) and emission lifetimes (τ) of **1**–**2** are around 10^−3^–10^−1^ and 10^2^–10^1^ ns respectively, while those parameters for [Os(C^C^C)(N^N)(CO)]^+^ are around 10^−4^–10^−2^ and 1–6 μs respectively^30^. Similar to Os(C^C^C)(N^N)(CO)]^+^, these photophysical parameters for **1**–**2** are sensitive to the change of C^C^C and N^N, revealing that the emissive state involve both the C^C^C and N^N moieties. For example, in both **1**–**2** and [Os(C^C^C)(N^N)(CO)]^+^, blue-shift on emission maxima, higher emission quantum yield, and longer excited state lifetime are observed when changing the N^N from 2,2-bipyridine to 1,10-phenanthroline^30^. The resemblance of the excitation profiles to the absorption spectra signifies that the emissions originate from the energy dissipation of the d_π_(Ir^III^) → π*(N^N) MLCT transitions. Interestingly, similar conclusion has been made on the nature of the emissive excited states in [Os(C^C^C)(N^N)(CO)]^+^ 30. The emission profile for [Ir(C^1^^C^C^1^)_2_]^+^ is highly structured and the emission maxima (378 and 398 nm) are not sensitive to the change of solvent, therefore these emissions are assigned as π → π* (C^C^C) ^3^IL emissions.

### Theoretical Calculations

Time-dependent density functional theory (TD-DFT) calculations have been performed on modeling complexes [Ir(^Me^C^1^^C^C^1Me^)(bpy)(H)]^+^ (**1a’**) and [Ir(^Me^C^2^^C^C^2Me^)(bpy)(H)]^+^ (**2a’**), in which their metal cores are the same as **1a** and **2a** but the butyl chains on the C^C^C are replaced by methyl groups to reduce computational cost. The ground-state structures of **1a’** and **2a’** have been optimized at the DFT level (functional = PBE0)^45^,^46^ without symmetry constrain. The conductor-like screening model (COSMO)^47^ has been applied to account for solvent effects upon the electronic transition. All the optimized geometries are in satisfactory agreement with their crystal structures. For example, the Ir–C_NHC_ and Ir–C_Ph_ bond distances calculated for **1a’** (2.05–2.06 and 1.97 Å respectively) are similar to those for **1a** determined by X-ray crystallography (Ir–C_NHC_: 2.049(3) and 2.055(3) Å; Ir–C_Ph_: 1.975(3) Å).

**Figure 6:**
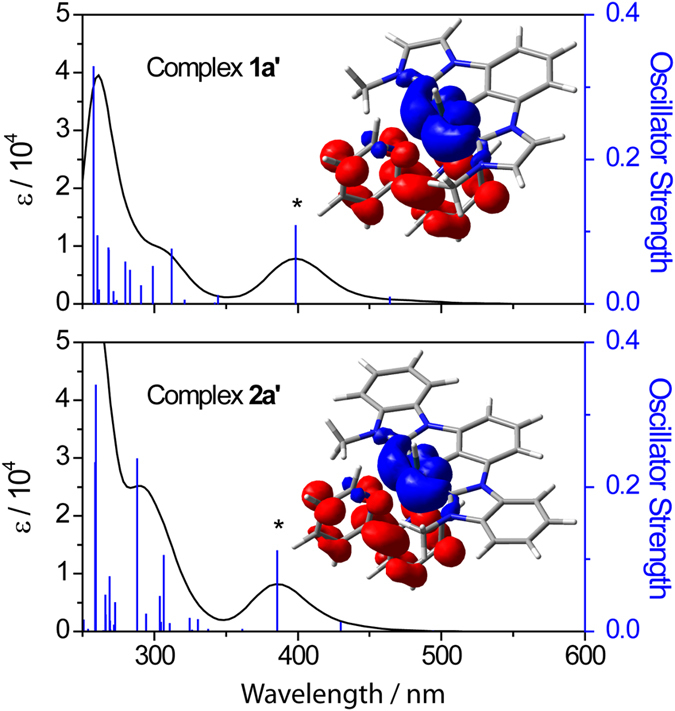
TD-DFT calculated absorption spectrum for model complexes 1a’ and 2a’ in CH_3_CN. Excitation energies and oscillator strengths are shown by the blue vertical lines; spectrum (in black) is convoluted with a Gaussian function having a full width at half-maximum of 3000 cm^−1^. Inserts show the electronic difference density plots for **1a’** and **2a’** at the vertical transitions marked with * (isodensity value = 0.002 au; charge accumulation and depletion are represented in red and blue respectively).

The excitation energies and oscillator strengths for the calculated vertical transitions with λ > 360 nm are summarized in Table 5. Table 6 summarized the compositions of the molecular orbitals (MOs) which are involved in the lowest-energy electronic transitions in these complexes. Figure 6 depicts the simulated absorption spectra. It is noted that the calculated lowest-energy dipole allowed transitions (λ > 360 nm) mainly originate from the HOMO–1 → LUMO and HOMO–2 → LUMO transitions. The HOMOs–1 and HOMOs–2 have higher Ir contribution (27–59%) than that in LUMOs (3–4%), whereas the LUMOs have higher N^N contribution (93%) than those in HOMOs–1 and HOMOs–2 (3–15%), therefore the transitions contain some Ir → π*(N^N) MLCT character. This finding is consistent with the spectroscopic observation that a red-shift in absorption energy is observed when N^N is changed from Me_2_bpy to bpy, and from phen to dpq. Besides, the contribution of C^C^C to both the HOMOs–1 and HOMOs–2 are not low (27–69%), suggesting that the C^C^C ligands contribute significantly to the d_π_(Ir^III^) levels. The electronic difference density plots for **1a’** and **2a’** in their lowest-energy excited state (Figure 6, generated by taking the difference in the excited-state electron density and ground-state electron density) clearly show that electronic charge is depleted from the Ir center and accumulated at the N^N moiety. The emissions from complexes **1**–**2** are thus believed to be originated from the triplet d_π_(Ir^III^) → π*(N^N) MLCT states.

## Conclusion

In this work a series of emissive Ir(III) hydrido complexes bearing the NHC-derived tridentate C^C^C pincer ligands and aromatic diimines have been prepared. This joint experimental and theoretical study reveals that the lowest-energy absorptions associated with these complexes arise from a d_π_(Ir^III^) → π*(N^N) MLCT transition, where the C^C^C ligands contribute significantly to the d_π_(Ir^III^) level. It is therefore evident that the C^C^C ligands can modulate the photophysical properties via the formation of the hybrid [Ir + C^C^C] molecular orbitals, and this work highlights the opportunities of using NHC-derived ligands to modulate the photophysics of a [M(N^N)] core.

## Methods

### General Procedure

**Figure 7:**
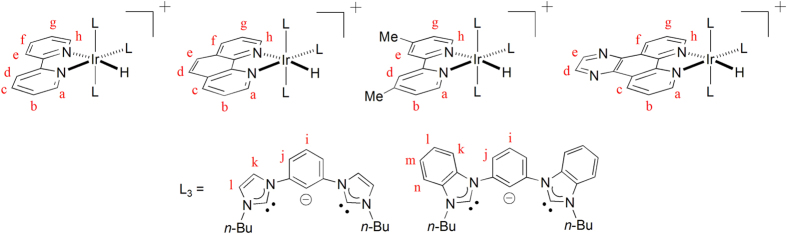
Labeling scheme for H and C atoms in 1–2.

All reactions were performed under an argon atmosphere using standard Schlenk techniques unless otherwise stated. All reagents and solvents were used as received. The C^C^C ligand precursors, i.e. benzene-bridged bisimidazolium bromide^48^, and [Ir(1,5-cod)Cl]_2_ (cod = 1,5-cyclooctadiene)^49^, were prepared according to literature methods. [Ir(C^C^C)(CH_3_CN)(Br)(H)] were synthesized analogously to the corresponding known complexes [Ir(^Ar^C^C^C^Ar^)(CH_3_CN)(Cl)(H)] and [Ir(^Me^C^1^^C^C^1Me^)(CH_3_CN)(I)(H)]^23^,^24^. ^1^H, ^13^C{^1^H}, DEPT-135, ^1^H–^1^H COSY, and ^1^H–^13^C HSQC NMR spectra were recorded on Bruker 400 DRX FT-NMR spectrometer. Figure 7 depicts the labeling scheme for the H and C atoms. Peak positions were calibrated with solvent residue peaks as internal standard. Electrospray mass spectrometry was performed on a PE-SCIEX API 3000 triple quadrupole mass spectrometer. Infrared spectra were recorded as KBr plates on an Avatar 360 FTIR spectrometer. UV–visible spectra were recorded on a Shimadzu UV-1700 spectrophotometer. Elemental analyses were done on an Elementar Vario Micro Cube carbon–hydrogen–nitrogen elemental micro-analyzer. Cyclic voltammetry was performed with a CH Instrument model 600C series electrochemical analyzer/workstation. All the electrochemical measurements were performed in CH_3_CN solution with [*n*-Bu_4_N]PF_6_ (0.1 M) as supporting electrolyte at room temperature. The glassy-carbon working electrode was polished with 0.05 *μ*m alumina on a microcloth, sonicated for 5 min in deionized water, and rinsed with CH_3_CN before use. An Ag/AgNO_3_ (0.1 M in CH_3_CN) electrode was used as reference electrode, with a platinum wire as the counter electrode. All solutions were degassed with nitrogen before experiments. The *E*_1/2_ value of the ferrocenium/ferrocene couple (Cp_2_Fe^+/0^) measured in the same solution was used as an internal reference. Steady-state emission spectra were obtained on a Jobin Yvon Fluorolog-3-TCSPC spectrophotometer. Sample and standard solutions were degassed with at least three freeze-pump-thaw cycles. The emission quantum yields for complexes **1**–**2** were measured by the method of Demas and Crosby^50^ with [Ru(bpy)_3_](PF_6_)_2_ in degassed CH_3_CN as standard (Φ_r_ = 0.062), whereas that for [Ir(C^1^^C^C^1^)_2_]^+^ was measured with quinine sulphate in 0.1M H_2_SO_4_ as standard (Φ_r_ = 0.58)^51^. The emission quantum yields were calculated by Φ_s_ = Φ_r_(*B*_r_/*B*_s_)(*n*_s_/*n*_r_)^2^(*D*_s_/*D*_r_), where the subscripts s and r refer to sample and reference standard solution, respectively, *n* is the refractive index of the solvents, *D* is the integrated intensity, and Φ is the luminescence quantum yield. The quantity *B* is calculated by *B* = 1 - 10^*−AL*^, where *A* is the absorbance at the excitation wavelength and *L* is the optical path length^52^.

### [Ir(C^C^C)(N^N)H](ClO_4_), 1–2(ClO_4_)

A mixture of [Ir(1,5-cod)Cl]_2_ (0.10 mmol), benzene bridged bisimidazolium or bisbenzimidazolium bromide (0.20 mmol), and Cs_2_CO_3_ (0.43 mmol) was refluxed in CH_3_CN (30 ml) for 16 h. Upon cooling to room temperature, the solvent was removed by reduced pressure and the residue was extracted with CH_2_Cl_2_. The [Ir(C^C^C)(CH_3_CN)(Br)(H)] obtained from this extract was used for the synthesis of **1**–**2** without further purification. A mixture of [Ir(C^C^C)(CH_3_CN)(Br)(H)] (0.15 mmol) and diimine (0.5 mmol) was refluxed in ethylene glycol for 3 h. Upon cooling to room temperature, the resultant solution was added to a saturated NaClO_4_ solution to give brown solids. The crude product was eluted by column chromatography (neutral alumina, 9:1 (v/v) CH_2_Cl_2_/CH_3_CN as eluent) as a yellow band. After removal of solvent, the yellow solid was recrystallized by slow diffusion of Et_2_O into CH_3_CN solution to give yellow crystals.

### Complex 1a

Yield: 0.06 g, 50%. Anal. Calcd for C_30_H_34_N_6_Ir(ClO_4_): C, 46.78; H, 4.45; N, 10.91. Found: C, 46.70; H, 4.51; N, 10.88. ^1^H NMR (400 MHz, CD_3_CN): *δ* –20.46 (s, 1H, Ir−H), 0.58–0.99, 1.18–1.32 (m, 14H, C_3_H_7_ of *n*-Bu); 3.17–3.22 (m, 4H, CH_2_ of *n*-Bu); 7.05 (d, 2H, *J* = 2.1 Hz, H_l_); 7.16–7.16 (m, 1H, H_g_); 7.22–7.29 (m, 3H, H_i_ + H_j_); 7.41 (d, 1H, *J* = 5.2 Hz, H_h_); 7.65–7.69 (m, 1H, H_b_); 7.74 (d, 2H, *J* = 2.1 Hz, H_k_); 7.88 (td, 1H, *J* = 8.0, 1.6 Hz, H_f_); 8.21 (td, 1H, *J* = 8.0, 1.6 Hz, H_c_); 8.38 (d, 1H, *J* = 8.0 Hz, H_e_); 8.55 (d, 1H, *J* = 8.0 Hz, H_d_); 9.67 (d, 1H, *J* = 5.2 Hz, H_a_). ^13^C NMR (100 MHz, CD_3_CN): *δ* 13.7, 20.2, 34.5, 50.1 (*n*-Bu); 108.3 (C_j_); 117.0 (C_k_); 121.6 (C_l_); 122.9 (C_i_); 124.6 (C_e_); 125.3 (C_d_); 127.7 (C_g_); 129.0 (C_b_); 138.2 (C_c_); 138.3 (C_f_); 142.9 (Ir−C_Ph_); 146.1 (Quaternary C in C^1^^C^C^1^); 151.7 (C_h_); 156.9, 157.0 (Quaternary C in bpy); 157.2 (C_a_); 167.9 (Ir−C_carbene_). IR (KBr, cm^−1^): ν_Ir−H_ = 2189, ν_Cl–O_ = 1086. ESI-MS: *m/z* 670 [M^+^].

### Complex 1b

Yield: 0.06 g, 50%. Anal. Calcd for C_32_H_34_N_6_Ir(ClO_4_): C, 48.39; H, 4.31; N, 10.58. Found: C, 48.08; H, 4.36; N, 10.40. ^1^H NMR (400 MHz, CD_3_CN): *δ* –20.27 (s, 1H, Ir−H), 0.14–0.51, 0.89–1.06 (m, 14H, C_3_H_7_ of *n*-Bu); 2.92–3.15 (m, 4H, CH_2_ of *n*-Bu); 6.98 (d, 2H, *J* = 2.0 Hz, H_l_); 7.21–7.37 (m, 3H, H_i_ + H_j_); 7.51 (dd, 1H, *J* = 8.0, 5.0 Hz, H_g_); 7.74 (d, 2H, *J* = 2.0 Hz, H_k_); 7.77 (d, 1H, *J* = 5.0 Hz, H_h_); 8.04 (dd, 1H, *J* = 8.0, 5.0 Hz, H_b_); 8.12 (d, 1H, *J* = 8.8 Hz, H_e_); 8.22 (d, 1H, *J* = 8.8 Hz, H_d_); 8.44 (d, 1H, *J* = 8.0 Hz, H_f_); 8.78 (d, 1H, *J* = 8.0 Hz, H_c_); 9.97 (d, 1H, *J* = 5.0 Hz, H_a_). ^13^C NMR (100 MHz, CD_3_CN): *δ* 13.6, 20.0, 34.4, 50.1 (*n*-Bu); 108.4 (C_j_); 117.1 (C_k_); 121.6 (C_l_); 123.2 (C_i_); 126.4 (C_g_); 127.8 (C_b_); 128.8 (C_d_); 128.9 (C_e_); 132.1, 132.6 (Quaternary C in phen); 137.4 (C_c_); 137.6 (C_f_); 142.6 (Ir−C_Ph_); 146.4 (Quaternary C in C^1^^C^C^1^); 148.4, 148.8 (Quaternary C in phen); 152.6 (C_h_); 157.3 (C_a_); 168.1 (Ir−C_carbene_). IR (KBr, cm^−1^): ν_Ir−H_ = 2179, ν_Cl–O_ = 1094. ESI-MS: *m/z* 695 [M^+^].

### Complex 1c

Yield: 0.05 g, 40%. Anal. Calcd for C_32_H_38_N_6_Ir(ClO_4_): C, 48.14; H, 4.80; N, 10.53. Found: C, 48.44; H, 5.08; N, 10.45. ^1^H NMR (400 MHz, CD_3_CN): *δ* –20.55 (s, 1H, Ir−H), 0.58–0.98, 1.19–1.38 (m, 14H, C_3_H_7_ of *n*-Bu); 2.39 (s, 3H, CH_3_ of Me_2_bpy); 2.61 (s, 3H, CH_3_ of Me_2_bpy); 3.26 (t, 4H, *J* = 7.8 Hz, CH_2_ of *n*-Bu); 6.96 (d, 1H, *J* = 5.6 Hz, H_g_); 7.05 (d, 2H, *J* = 2.1 Hz, H_l_); 7.20 (d, 1H, *J* = 5.6 Hz, H_h_); 7.21–7.29 (m, 3H, H_i_ + H_j_); 7.50 (d, 1H, *J* = 5.6 Hz, H_b_); 7.73 (d, 2H, *J* = 2.1 Hz, H_k_); 8.23 (s, 1H, H_e_); 8.39 (s, 1H, H_d_); 9.46 (d, 1H, *J* = 5.6 Hz, H_a_). ^13^C NMR (100 MHz, CD_3_CN): *δ* 13.7, 20.3, 34.5, 50.1 (*n*-Bu); 21.1, 21.3 (CH_3_ of Me_2_bpy); 108.2 (C_j_); 117.0 (C_k_); 121.5 (C_l_); 122.7 (C_i_); 125.2 (C_e_); 125.8 (C_d_); 128.4 (C_g_); 129.6 (C_b_); 143.4 (Ir−C_Ph_); 146.1 (Quaternary C in C^1^^C^C^1^); 150.5, 150.7 (Quaternary C in Me_2_bpy); 150.9 (C_h_); 156.6 (C_a_); 156.8, 156.9 (Quaternary C in Me_2_bpy); 168.4 (Ir−C_carbene_). IR (KBr, cm^−1^): ν_Ir−H_ = 2159, ν_Cl–O_ = 1107. ESI-MS: *m/z* 699 [M^+^].

### Complex 1d

Yield: 0.06 g, 45%. Anal. Calcd for C_34_H_34_N_8_Ir(ClO_4_): C, 48.25; H, 4.05; N, 13.24. Found: C, 48.47; H, 4.28; N, 13.06. ^1^H NMR (400 MHz, CD_3_CN): *δ* –20.29 (s, 1H, Ir−H), 0.27–0.75, 1.08–1.21 (m, 14H, C_3_H_7_ of *n*-Bu); 2.91–3.22 (m, 4H, CH_2_ of *n*-Bu); 7.00 (d, 2H, *J* = 2.1 Hz, H_l_); 7.24–7.37 (m, 3H, H_i_ + H_j_); 7.65 (dd, 1H, *J* = 8.2, 5.2 Hz, H_g_); 7.76 (d, 2H, *J* = 2.0 Hz, H_k_); 7.87–7.88 (m, 1H, H_h_); 8.18 (dd, 1H, *J* = 8.2, 5.2 Hz, H_b_); 9.18 (d, 1H, *J* = 2.1 Hz, H_e_); 9.22 (d, 1H, *J* = 2.1 Hz, H_d_); 9.41 (dd, 1H, *J* = 8.2, 1.2 Hz, H_f_); 9.75 (dd, 1H, *J* = 8.2, 1.2 Hz, H_c_); 10.09 (dd, 1H, *J* = 5.2, 1.2 Hz, H_a_). ^13^C NMR (100 MHz, CD_3_CN): *δ* 13.5, 20.1, 34.3, 50.2 (*n*-Bu); 108.5 (C_j_); 117.2 (C_k_); 121.7 (C_l_); 123.3 (C_i_); 127.6 (C_g_); 128.9 (C_b_); 130.8, 131.5 (Quaternary C in dpq); 134.0 (C_c_); 134.2 (C_f_); 140.5, 140.5 (Quaternary C in dpq); 142.2 (Ir−C_Ph_); 146.3 (Quaternary C in C^1^^C^C^1^); 147.7, 147.8 (C_d_ + C_e_); 149.7, 150.0 (Quaternary C in dpq); 153.9 (C_h_); 158.5 (C_a_); 167.8 (Ir−C_carbene_). IR (KBr, cm^−1^): ν_Ir−H_ =2131, ν_Cl–O_ = 1108. ESI-MS: *m/z* 747 [M^+^].

### Complex 2a

Yield: 0.07 g, 50%. Anal. Calcd for C_38_H_38_N_6_Ir(ClO_4_): C, 52.44; H, 4.40; N, 9.66. Found: C, 52.45; H, 4.38; N, 9.46. ^1^H NMR (400 MHz, CD_3_CN): *δ* –19.50 (s, 1H, Ir−H), 0.40–1.07, 1.23–1.47 (m, 14H, C_3_H_7_ of *n*-Bu); 3.30–3.59 (m, 4H, CH_2_ of *n*-Bu); 7.07 (t, 1H, *J* = 6.4 Hz, H_g_); 7.28–7.35 (m, 2H, H_m_); 7.37–7.50 (m, 6H, H_h_ + H_i_ + H_l_ + H_n_); 7.71–7.74 (m, 1H, H_b_); 7.77–7.87 (m, 3H, H_j_ + H_f_); 8.21 (d, 2H, *J* = 8.0 Hz, H_k_); 8.27–8.31 (m, 1H, H_c_); 8.38 (d, 1H, *J* = 8.2 Hz, H_e_); 8.60 (d, 1H, *J* = 8.2 Hz, H_d_); 9.51 (d, 1H, *J* = 5.3 Hz, H_a_). ^13^C NMR (100 MHz, CD_3_CN): *δ* 13.8, 20.4, 33.3, 47.5 (*n*-Bu); 109.7 (C_j_); 111.9 (C_l_/C_n_); 112.2 (C_k_); 123.6 (C_i_); 123.9 (C_m_); 124.6 (C_e_); 124.7 (C_l_/C_n_); 125.5 (C_d_); 127.9 (C_g_); 129.3 (C_b_); 133.0, 135.6 (Quaternary C in C^2^^C^C^2^); 138.7 (C_f_); 138.8 (C_c_); 142.9 (Ir−C_Ph_); 146.6 (Quaternary C in C^2^^C^C^2^); 152.2 (C_h_); 156.6 (Quaternary C in bpy); 157.2 (C_a_); 157.3 (Quaternary C in bpy); 180.1 (Ir−C_carbene_). IR (KBr, cm^−1^): ν_Ir−H_ = 2126, ν_Cl–O_ = 1075 ESI-MS: *m/z* 771 [M^+^].

### Complex 2b

Yield: 0.05 g, 40%. Anal. Calcd for C_40_H_38_N_6_Ir(ClO_4_): C, 53.71; H, 4.28; N, 9.40. Found: C, 53.75; H, 4.30; N, 9.45. ^1^H NMR (400 MHz, CD_3_CN): *δ* –19.21 (s, 1H, Ir−H), 0.19–0.35, 0.38–0.45, 0.52–0.67 (m, 14H, C_3_H_7_ of *n*-Bu); 3.26–3.46 (m, 4H, CH_2_ of *n*-Bu); 7.26–7.39 (m, 4H, H_l_/H_m_ + H_n_); 7.39–7.48 (m, 3H, H_g_ + H_m_/H_l_); 7.52 (t, 1H, *J* = 8.0 Hz, H_i_); 7.81 (d, 2H, *J* = 5.1 Hz, H_f_); 7.87 (d, 2H, *J* = 8.0 Hz, H_j_); 8.08–8.18 (m, 2H, H_b_ + H_e_); 8.21–8.29 (m, 3H, H_d_ + H_k_); 8.41 (d, 1H, *J* = 8.2 Hz, H_h_); 8.88 (d, 1H, *J* = 8.2 Hz, H_c_); 9.96 (d, 1H, *J* = 8.2 Hz, H_a_). ^13^C NMR (100 MHz, CD_3_CN): *δ* 13.8, 20.3, 33.3, 47.6 (*n*-Bu); 109.8 (C_j_); 111.9 (C_l_/C_m_/C_n_); 112.3 (C_k_); 123.9 (C_i_); 124.0 (C_l_/C_m_/C_n_); 124.8 (C_l_/C_m_/C_n_); 126.7 (C_g_); 128.1 (C_b_); 128.8 (C_d_); 129.1 (C_e_); 132.2, 132.7 (Quaternary C in phen); 133.1, 135.7 (Quaternary C in C^2^^C^C^2^); 137.9 (C_h_); 138.1 (C_c_); 142.6 (Ir−C_Ph_); 146.9 (Quaternary C in C^2^^C^C^2^); 148.3, 148.9 (Quaternary C in phen); 153.2 (C_f_); 157.4 (C_a_); 180.4 (Ir−C_carbene_). IR (KBr, cm^−1^): ν_Ir−H_ = 2129, ν_Cl–O_ = 1090. ESI-MS: *m/z* 795 [M^+^].

### Complex 2c

Yield: 0.05 g, 40%. Anal. Calcd for C_40_H_42_N_6_Ir(ClO_4_): C, 53.47; H, 4.71; N, 9.35. Found: C, 53.53; H, 4.77; N, 9.57. ^1^H NMR (400 MHz, CD_3_CN): *δ* –19.48 (s, 1H, Ir−H), 0.60–0.97, 1.34–1.47 (m, 14H, C_3_H_7_ of *n*-Bu); 2.33 (s, 3H, CH_3_ of Me_2_bpy); 2.67 (s, 3H, CH_3_ of Me_2_bpy); 3.39–3.67 (m, 4H, CH_2_ of *n*-Bu); 6.90 (d, 1H, *J* = 5.7 Hz, H_g_); 7.25 (d, 1H, *J* = 5.7 Hz, H_h_); 7.28–7.38 (m, 2H, H_m_); 7.40–7.50 (m, 5H, H_i_ + H_l_ + H_n_); 7.54–7.65 (m, 1H, H_b_); 7.81 (d, 2H, *J* = 8.0 Hz, H_j_); 8.17–8.35 (m, 3H, H_e_ + H_k_); 8.46 (s, 1H, H_d_); 9.42 (d, 1H, *J* = 5.6 Hz, H_a_). ^13^C NMR (100 MHz, CD_3_CN): *δ* 13.8, 20.5, 33.4, 47.6 (*n*-Bu); 21.1, 21.3 (CH_3_ of Me_2_bpy); 109.6 (C_j_); 111.9 (C_l_/C_n_); 112.2 (C_k_); 123.5 (C_i_); 123.9 (C_m_); 124.7 (C_l_/C_n_); 125.3 (C_e_); 126.0 (C_d_); 128.6 (C_g_); 130.0 (C_b_); 133.0, 135.7 (Quaternary C in C^2^^C^C^2^); 143.4 (Ir−C_Ph_); 146.7 (Quaternary C in C^2^^C^C^2^); 151.1, 151.3 (Quaternary C in Me_2_bpy); 151.3 (C_h_); 156.6 (Quaternary C in Me_2_bpy); 156.7 (C_a_); 157.1 (Quaternary C in Me_2_bpy); 180.6 (Ir−C_carbene_). IR (KBr, cm^−1^): ν_Ir−H_ = 2133, ν_Cl–O_ = 1094. ESI-MS: *m/z* 799 [M^+^].

### Complex 2d

Yield: 0.06 g, 40%. Anal. Calcd for C_42_H_38_N_8_Ir(ClO_4_): C, 53.30; H, 4.05; N, 11.84. Found: C, 53.41; H, 4.25; N, 11.94. ^1^H NMR (400 MHz, CD_3_CN): *δ* –19.22 (s, 1H, Ir−H), 0.23–0.79, 1.18–1.48 (m, 14H, C_3_H_7_ of *n*-Bu); 3.17–3.61 (m, 4H, CH_2_ of *n*-Bu); 7.28–7.46 (m, 6H, H_l_ + H_m_ + H_n_); 7.54 (t, 1H, *J* = 8.0 Hz, H_i_); 7.59 (dd, 1H, *J* = 8.2, 5.2 Hz, H_g_); 7.89 (d, 2H, *J* = 8.0 Hz, H_j_); 7.91–7.93 (m, 1H, H_h_); 8.16–8.35 (m, 3H, H_b_ + H_k_); 9.18 (d, 1H, *J* = 2.1 Hz, H_e_); 9.24 (d, 1H, *J* = 2.1 Hz, H_d_); 9.37 (dd, 1H, *J* = 8.2, 1.4 Hz, H_f_); 9.85 (dd, 1H, *J* = 8.2, 1.3 Hz, H_c_); 10.11 (dd, 1H, *J* = 5.2, 1.3 Hz, H_a_). ^13^C NMR (100 MHz, CD_3_CN): *δ* 13.7, 20.4, 33.3, 47.7 (*n*-Bu); 109.9 (C_j_), 112.0 (C_l_/C_m_/C_n_); 112.3 (C_k_); 124.1 (C_i_); 124.1, 124.9 (C_l_/C_m_/C_n_); 127.8 (C_g_); 129.2 (C_b_); 130.9, 131.6 (Quaternary C in dpq); 133.1 (Quaternary C in C^2^^C^C^2^); 134.5 (C_c_), 134.6 (C_f_), 135.7 (Quaternary C in C^2^^C^C^2^); 140.4, 140.5 (Quaternary C in dpq); 142.3 (Ir−C_Ph_), 146.9 (Quaternary C in C^2^^C^C^2^), 147.8 (C_e_); 147.9 (C_d_); 149.5, 150.2 (Quaternary C in dpq); 154.5 (C_h_); 158.6 (C_a_); 180.1 (Ir−C_carbene_). IR (KBr, cm^−1^): ν_Ir−H_ = 2130, ν_Cl–O_ = 1097. ESI-MS: *m/z* 847 [M^+^].

### X-ray Crystallography

X-ray diffraction data for **1a**(ClO_4_), **2a**(ClO_4_), and [**2b**(ClO_4_)]_3_·CH_3_CN were collected on an Oxford Diffraction Gemini S Ultra X-ray single crystal diffractometer with Cu K*α* radiation (λ = 1.54178 Å) at 133 K. The data were processed using CrysAlis^53^. The structures were solved by Patterson and Fourier methods and refined by full-matrix least-squares based on *F*^2^ with program SHELXS-97 and SHELXL-97^54^ within WinGX^55^. All non-hydrogen atoms were refined anisotropically in the final stage of least-squares refinement. The positions of H atoms were calculated based on riding mode with thermal parameters equal to 1.2 times that of the associated C atoms. CCDC 1416088–1416090 contain the supplementary crystallographic data for this paper, which can be obtained free of charge from The Cambridge Crystallographic Data Centre via www.ccdc.cam.ac.uk/data_request/cif.

### Computational Methodology

DFT calculations were performed on model complexes [Ir(^Me^C^1^^C^C^1Me^)(bpy)(H)]^+^ (**1a’**) and [Ir(^Me^C^2^^C^C^2Me^)(bpy)(H)]^+^ (**2a’**). Their electronic ground states were optimized without symmetry constrain using the density functional PBE0^45^,^46^. The def2-SVP basis sets were used for the H, C, and N atoms, while the def2-TZVP(-f) basis sets were used for the Ir atoms^56^. Zero-order regular approximation (ZORA) was employed to account for relativistic effects. Tight SCF convergence (10^−8^au) was used for all calculations. The vertical transition energies for these model complexes in CH_3_CN were computed at their respective gas-phase optimized ground-state geometries using time-dependent-DFT (TD-DFT) method with the same density functional and basis sets in the geometry optimizations. The combination of the resolution of the identity and the “chain of spheres exchange” algorithms (RIJCOSX)^57^ was used to accelerate all DFT and TD-DFT calculations with the use of appropriate auxiliary basis sets. The conductor-like screening model (COSMO)^47^ was used to account for solvent effects upon the electronic transition. All the calculations were performed using the ORCA software package (version 3.0.2)^58^.

## Additional Information

**How to cite this article**: Chung, L.-H. *et al*. Luminescent Iridium(III) Complexes Supported by N-Heterocyclic Carbene-based C^C^C-Pincer Ligands and Aromatic Diimines. *Sci. Rep.* **5**, 15394; doi: 10.1038/srep15394 (2015).

## Figures and Tables

**Table 1:** Selected Bond Lengths (Å) and Angles (deg) for 1a, 2a, and 2b.

complex	1a	2a	2b
Ir–C_NHC_	2.049(3), 2.055(3)	2.044(4), 2.046(4)	[2.052(5), 2.056(5)]; [2.045(5), 2.049(5)]; [2.043(5), 2.044(5)]
Ir–C_Ph_	1.975(3)	1.959(4)	1.986(5); 1.982(5); 1.980(5)
Ir–N_N^N_ (trans to Ph)	2.130(2)	2.134(3)	2.140(4); 2.134(4); 2.149(4)
Ir–N_N^N_ (trans to H)	2.144(2)	2.154(3)	2.135(5); 2.148(4); 2.141(5),
C_NHC_–Ir–C_Ph_	77.22(11), 77.86(11)	78.30(15), 78.74(15)	[77.7(2), 77.8(2)]; [77.8(2), 77.9(2)]; [77.7(2), 78.7(2)]
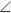 Ph/NHC	7.90, 16.96	5.06, 7.35	[4.86, 10.71]; [8.21, 8.35]; [3.35, 10.50]
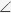 NHC/NHC	22.54	12.36	14.83; 16.41; 13.85

The angle between the rings (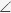
Ph/NHC or 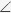
NHC/NHC) are calculated from all non-hydrogen atoms on the ring moiety). For **2b**, the crystal contains three crystallographically independent Ir complexes in an asymmetric unit; structural data from each complex are grouped in brackets and listed in the order of Ir(1), Ir(2), and Ir(3).

**Table 2:** Electrochemical Data.

Complex	*E*_1/2_/V vs Cp_2_Fe^+/0^	
	reduction	oxidation
**1a**	−1.93	*E*_pa_ = 0.7
**1b**	−1.95	*E*_pa_ = 0.71
**1c**	−2.00	*E*_pa_ = 0.69
**1d**	−1.83	*E*_pa_ = 0.70
**2a**	−1.87	*E*_pa_ = 0.76
**2b**	−1.87	*E*_pa_ = 0.76
**2c**	−1.95	*E*_pa_ = 0.73
**2d**	−1.73	*E*_pa_ = 0.77
Ir(C^1^^C^C^1^)_2_]^+^	—	0.71

Supporting electrolyte: 0.1 M [Bu_4_N]PF_6_ in CH_3_CN. *E*_1/2_ = (*E*_pc_ + *E*_pa_)/2 at 298 K for reversible couples. Anodic peak potential (*E*_pa_) at scan rate of 100 mV s^−1^ are recorded for irreversible oxidation wave.

**Table 3:** UV–Visible Absorption Data.

complex	λ_max_/nm (ε_max_/dm^3^ mol^−1^ cm^−1^)
Solvent = CH_3_CN	
**1a**	241 (sh, 36670), 283 (26380), 308 (sh, 14980), 319 (sh, 11720), 374 (4240), 439 (sh, 1620)
**1b**	246 (33910), 260 (30980), 281 (sh, 21780), 308 (10680), 320 (11250), 370 (5460), 455 (sh, 1720)
**1c**	242 (sh, 37920), 281 (27830), 304 (sh, 16760), 321 (sh, 13090), 371 (4640), 430 (sh, 1950)
**1d**	250 (sh, 57990), 256 (58720), 288 (37050), 319 (14630), 374 (7180), 459 (sh, 2210)
**2a**	247 (63480), 271 (44590), 285 (sh, 35650), 309 (25000), 321 (22070), 370 (sh, 5240), 422 (sh, 1710)
**2b**	248 (57050), 269 (46670), 286 (sh, 30100), 312 (sh, 17530), 321 (19930), 376 (sh, 5090), 430 (sh, 1720)
**2c**	247 (64180), 270 (48540), 307 (26390), 322 (23220), 369 (sh, 5520), 425 (sh, 1650)
**2d**	248 (77070), 256 (74100), 285 (47240), 320 (23660), 365 (sh, 7060), 453 (sh, 1660)
**[Ir(C**^**1**^**^C^C**^**1**^)_**2**_]^**+**^	279 (18810), 305 (15410), 318 (15270)
Solvent = CH_2_Cl_2_	
**1a**	240 (sh, 40380), 285 (30500), 308 (sh, 15420), 318 (sh, 12550), 351 (4400), 384 (4510), 447 (sh, 1770)
**1b**	247 (sh, 34120), 262 (33960), 280 (sh, 22810), 309 (10810), 321 (11075), 375 (5690), 472 (sh, 1750)
**1c**	242 (sh, 40650), 283 (30940), 306 (sh, 16480), 322 (13600), 378 (4670), 422 (sh, 1980)
**1d**	252 (sh, 60080), 257 (61150), 289 (36820), 320 (14480), 381 (7310), 467 (sh, 2280)
**2a**	248 (67690), 272 (48350), 282 (sh, 45580), 309 (25340), 321 (23470), 375 (sh, 5460), 441 (sh, 1560)
**2b**	249 (65520), 269 (56000), 282 (sh, 40080), 311 (sh, 19100), 322 (22270), 385 (sh, 5890), 448 (sh, 1680)
**2c**	249 (63520), 272 (49670), 308 (25150), 323 (23440), 372 (sh, 5490), 431 (sh, 1820)
**2d**	249 (80990), 257 (76520), 286 (49670), 321 (23940), 379 (7330), 465 (sh, 1940)
**[Ir(C**^**1**^**^C^C**^**1**^)_**2**_]^**+**^	281 (19600), 305 (15680), 319 (15680)

**Table 4:** Emission Data for Complexes 1, 2, and [Ir(C^1^^C^C^1^)_2_]^+^ in solution at 298 K.

complex	λ_em_/nm	Quantum yield (Φ)	Lifetime (τ)/ns
Solvent = CH_3_CN			
**1a**	577	4.53 × 10^−3^	21
**1b**	563	3.50 × 10^−2^	244
**1c**	565	1.09 × 10^−2^	38
**1d**	580	7.97 × 10^−3^	30
**2a**	588	2.00 × 10^−3^	10
**2b**	575	8.99 × 10^−3^	51
**2c**	575	4.18 × 10^−3^	15
**2d**	604	2.45 × 10^−3^	11
**[Ir(C**^**1**^**^C^C**^**1**^)_**2**_]^**+**^	378	4.22 × 10^−1 *b*^	4663
	398		4802
	sh, 416		5001
Solvent = CH_2_Cl_2_			
**1a**	568	1.34 × 10^−2^	47
**1b**	553	1.19 × 10^−1^	790
**1c**	555	3.31 × 10^−2^	91
**1d**	567	3.82 × 10^−2^	121
**2a**	578	6.03 × 10^−3^	26
**2b**	560	4.92 × 10^−2^	181
**2c**	566	1.35 × 10^−2^	43
**2d**	584	1.16 × 10^−2^	51
**[Ir(C**^**1**^**^C^C**^**1**^)_**2**_]^**+**^	378	6.07 × 10^−1 *b*^	4906
	398		5094
	sh, 416		5298

Measurement conditions: Concentration = 3.0 × 10^−5^ M; λ_ex_ = 420 nm for **1**–**2**, 340 nm for [Ir(C^1^^C^C^1^)_2_]^+^. Quantum yields for complexes **1**–**2** and [Ir(C^1^^C^C^1^)_2_]^+^ were determined using [Ru(bpy)_3_]^2+^ and quinine sulphate as references, respectively.

**Table 5:** Calculated Vertical Transition Energies (λ > 360 nm) for 1a’ and 2a’ at the TD–DFT/COSMO level (Solvent = CH_3_CN).

complex	experimental λ_max_/cm^−1^ (ε_max_/dm^3^ mol^−1^ cm^−1^)	TD-DFT calculations	
		excitation energy/cm^−1^(oscillator strength)	contribution
**1a’**	22780 (sh, 1620)	21540 (0.0086)	88.8% HOMO–1 → LUMO
			7.41% HOMO–2 → LUMO
			1.30% HOMO → LUMO
	26740 (4240)	25100 (0.1077)	87% HOMO–2 → LUMO
			7.07% HOMO–1 → LUMO
**2a’**	23700 (sh, 1710)	23260 (0.0121)	89.6% HOMO–1 → LUMO
			5.12% HOMO–2 → LUMO
			3.02% HOMO–3 → LUMO
			1.13% HOMO–8 → LUMO
	27030 (sh, 5240)	25920 (0.1109)	86.6% HOMO–2 → LUMO
			6.11% HOMO–1 → LUMO
			2.09% HOMO–3 → LUMO

Excitations with oscillator strength <5 × 10^−3^ are omitted; solvent = CH_3_CN.

**Table 6:** Selected Molecular Orbital Compositions (%) for 1a’ and 2a’.

complex	MO	% composition			
		Ir	N^N	C^C^C	H_hydride_
**1a’**	HOMO–2	58.93	14.51	26.56	0.00
	HOMO–1	34.22	3.77	61.99	0.02
	HOMO	33.74	4.45	61.53	0.28
	LUMO	3.50	92.93	3.57	0.00
**2a’**	HOMO–3	10.46	1.13	88.41	0.00
	HOMO–2	56.27	13.54	30.19	0.00
	HOMO–1	27.45	3.35	69.20	0.01
	HOMO	27.81	3.80	68.07	0.32
	LUMO	3.41	92.87	3.72	0.00
